# Effect of *Carissa opaca* leaves extract on lipid peroxidation, antioxidant activity and reproductive hormones in male rats

**DOI:** 10.1186/1476-511X-12-90

**Published:** 2013-06-20

**Authors:** Sumaira Sahreen, Muhammad Rashid Khan, Rahmat Ali Khan, Naseer Ali Shah

**Affiliations:** 1 Botanical Sciences Division, Pakistan Museum of Natural History, Garden Avenue, Shakarparian, Islamabad, Pakistan; 2 Department of Biochemistry, Faculty of Biological Sciences, Quaid-i-Azam University, Islamabad, Pakistan; 3 Department of Biotechnology, Faculty of Biological Sciences, University of Science and Technology Bannu, Khyber Pakhtunkhwa, KPK 28100, Pakistan

**Keywords:** CCl_4_, *Carissa opaca*, TBARS, LH, DNA fragmentation

## Abstract

**Background:**

*Carissa opaca* leaves are traditionally used in the treatment of male dysfunction and hormonal disorder as well as in oxidative stress in Pakistan and Asia. The present study was designed to assess the protective effects of methanolic extract of *Carissa opaca* leaves (MLC) on carbon tetrachloride (CCl_4_)-induced reproductive stress in male rats and bioactive constituents responsible for the activity.

**Methods:**

CCl_4_ was induced in 42 male rats for eight weeks and checked the protective efficacy of methanolic extract of *Carissa opaca* leaves at various hormonal imbalances, alteration of antioxidant enzymes, DNA fragmentation levels and lipid peroxidation caused testicular fibrosis in testis while High performance Liquid Chromatography (HPLC) was used for detection of bioactive components.

**Results:**

HPLC characterization revealed the presence of isoquercitin , hyperoside , vitexin , myricetin and kaempherol. CCl_4_ caused significant alteration in the secretion of reproductive hormones. Activity of antioxidant enzymes viz; catalase, superoxide dimutase and phase II metabolizing enzymes including glutathione peroxidase, glutathione reductase and reduced glutathione was decreased while DNA fragmentation, hydrogen per oxide contents and thiobarbituric acid reactive substances (TBARS) were increased with CCl_4_ treatment. Co-administration of 100 mg/kg and 200 mg/kg b.w. MLC effectively ameliorated the alterations in the biochemical markers; hormonal and molecular levels.

**Conclusion:**

Protective effects of methanolic extract of *Carissa opaca* against CCl_4−_induced antioxidant and hormonal dysfunction which might be due to bioactive compound present in extract.

## Background

During the last decade, considerable attention was given to the involvement of oxygen free radicals in various diseases. There is no doubt that reactive oxygen species (ROS) play an important role in pathological changes in the liver, particularly in the case of alcoholic and toxic liver diseases [1]. Biological membranes are particularly prone to ROS effect. The peroxidation of unsaturated fatty acids in biological membranes leads to the decrease of membrane fluidity and the disruption of membrane integrity and function. Such peroxidation effect is implicated in serious pathological changes [2]. CCl_4_ is an industrial solvent causes tissue damages in various tissues of experimental animals. CCl_4_ requires bioactivation by phase I cytochrome P450 system to form reactive metabolic radicals. These free radicals can bind with polyunsaturated fatty acid (PUFA) of sperm membrane to generate lipid peroxides that are highly reactive, change enzyme activity and finally induce injury or necrosis [3,4]. Several endogenous protective mechanisms have evolved to limit ROS effect and the damage caused by them [5]. However, when this protection is not complete, or when the formation of ROS is excessive, additional protective mechanisms of dietary antioxidants may be of a great importance. Therefore, many natural and synthetic agents possessing antioxidative properties have been proposed to prevent and treat infertility and reproductive hormonal imbalance induced by oxidative stress [6]. There is increasing evidence of the protective role of hydroxy and polyhydroxy organic compounds, particularly from vegetables, fruits and some herbs.

Plants are well-known excellent perspectives for the discovery of new therapeutical products. In recent years, an ample interest has been developed in finding natural antioxidants from commonly available wild plants, fruits and vegetables that were generally mistreated [7,8] as well as an important role in detoxification of free radicals induced lung injuries and fibrosis in experimental animal’s model. *Carissa opaca* Stapf ex Hanes a 2–3 meter tall evergreen shrub containing glabrous fruits widely found in Pakistan [9]. Traditionally this plant is used for the treatment of asthma [10], hepatitis [11], diarrhea [12] and renal dysfunction [13]. The present study was conducted to examine the toxic upshots of CCl_4_ plus to compare the beneficial effects of plant extracts on reproductive hormonal disturbance and activity of antioxidant enzymes in various experimental groups. In this respect, several parameters regarding the testicular injury and fibrosis were studied.

## Methods and materials

### Plant collection

*C. opaca* leaves were collected in March 2011 from the Quaid-i-Azam University Islamabad, Pakistan, recognized by their local names and validated by Dr. Mir Ajab Khan, Department of Plant Sciences, Quaid-i-Azam University, Islamabad. A voucher specimen was deposited at the Herbarium of Pakistan Quaid-i-Azam University, Islamabad Pakistan for future reference.

### Extract preparation

The collected plant leaves were cleaned and dried under shade for fifteen days. Willy Mill of 60-mesh size was used to prepare powder of dried samples and then 5 kg powdered plant sample was extracted twice with 10 L of 95% methanol at 25°C for 48 h. For filtration Whatman No. 1 filter paper was used and then filtrate was concentrated on rotary evaporator (Panchun Scientific Co., Kaohsiung, Taiwan) under reduced pressure at 40°C and dry extract was stored at 4°C for further *in vivo* investigation.

### Phytochemical investigation

#### High performance liquid chromatogrhy (HPLC)

50 mg of fine powder was extracted with 6 ml of 25% hydrochloric acid and 20 ml methanol for 1 h. The obtained extract was filtered to a volumetric flask and diluted to 100 ml with methanol. 10 μl was injected into HPLC column (20RBAX ECLIPSE, XDB-C18, 5 μm; 4.6 × 150 mm, Agilent USA) with UV–VIS Spectra-Focus detector, injector-auto sampler. Solvent A (0.05% trifluoroacetic acid) and solvent B (0.038% trifluoroacetic acid in 83% acetonitrile (v/v) with the following gradient: 0–5 min, 15% B in A, 5–10 min, 70% B in A, 10–15 min, 70% B in A. The flow rate was 1 ml/min and injection volume was 10 μl. Various standard compounds including rutin, myricetin, vitexin, orientin, hyperoside, isovitexin, isoquercetin, luteolin, apigenin, kaempherol, and luteolin-7-glucoside were run for comparative detection and optimized. The calibration curves were defined for each compound in the range of sample quantity 0.02-0.5 μg. All samples were assayed in triplicate.

### Experimental plan

Six-week-old male Sprague Dawley rats weighing 180 ± 10 g were provided with food and water *ad libitum* and kept at 20–22°C on a 12-h light–dark cycle. All experimental procedures involving animals were conducted in accordance with the guidelines of National Institutes of Health (NIH guidelines). The study protocol were approved by Ethical committee of Quaid-i-Azam University Islamabad. The rats were acclimatized to laboratory condition for 7 days before commencement of experiment. For chronic toxicity eight week experiment was designed. 42 male albino rats were randomly divided into seven groups (6 rats of each group). Administration of CCl_4_ (0.5 ml/kg b.w., 20% CCl_4_/olive oil) was intraperitoneally (i.p.) twice a week for eight weeks. At the same time, the rats were administered individually silymarin (50 mg/kg b.w.) and extract (100, 200 mg/kg b.w.) orally twice a week for eight weeks.

### Experimental protocol

Following dosing plan was adapted for the study.

Group I: the normal control received only feed

Group II: Olive oil (0.5 ml/kg b.w., i.p.) + DMSO (0.5 ml/kg b.w. orally)

Group III: CCl_4_ twice a week (0.5 ml/kg b.w., i.p., 20% CCl_4_/olive oil)

Group IV: CCl_4_ twice a week (0.5 ml/kg b.w., i.p.) + sylimarin (50 mg/kg b.w., orally)

Group V: CCl_4_ twice a week (0.5 ml/kg b.w., i.p.) + MLC (100 mg/kg b.w., orally)

Group VI: CCl_4_ twice a week (0.5 ml/kg b.w., i.p.) + MLC (200 mg/kg b.w., orally)

At the end of eight weeks, after 24 h of the last treatment, Urine was collected and stored at −70°C for further analysis, and then animals were given chloroform anesthesia and dissected from ventral side. All the animals were sacrificed; blood was drawn prior to the excision of organ tissues. The serum was stored at −80°C after separation until it was assayed as described below. After taking blood the testis were removed and washed in ice cold saline. Subsequently, half of the organs were treated with liquid nitrogen and stored at −80°C for further enzymatic and DNA damage analysis while the other portion was processed for histology.

### Assesment of reproductive hormones and lipid profile of serum

Serum analysis of testicular hormones like FSH, LH, testosterone, prolactin and esteradiol were radioimmunoassayed by using Marseille Cedax 9 France Kits and Czch Republic Kits from Immunotech Company. Then again, lipid profile such as Triglycerides, total cholesterol, LDL and LDH waere estimated by using standard AMP diagnostic kits (Stattogger Strasse 31b 8045 Graz, Austria).

### Assessment of antioxidant enzymes

10% homogenate of tissue was prepared in 100 mM KH_2_PO_4_ buffer containing 1 mM EDTA (pH 7.4) and centrifuged at 12,000 × g for 30 min at 4°C. The supernatant was collected and used for the following parameters as described below.

### Catalase assay (CAT)

CAT activities were determined by the method of Chance and Maehly [14] with some modifications. The reaction solution of CAT activities contained: 2.5 ml of 50 mM phosphate buffer (pH 5.0), 0.4 ml of 5.9 mM H_2_O_2_ and 0.1 ml enzyme extract. Changes in absorbance of the reaction solution at 240 nm were determined after one minute. One unit of CAT activity was defined as an absorbance change of 0.01 as units/min.

### Peroxidase assay (POD)

Activities of POD were determined by the method of Chance and Maehly [14] with some modifications. The POD reaction solution contained: 2.5 ml of 50 mM phosphate buffer (pH 5.0), 0.1 ml of 20 mM guaiacol, 0.3 ml of 40 mM H_2_O_2_ and 0.1 ml enzyme extract. Changes in absorbance of the reaction solution at 470 nm were determined after one minute. One unit of POD activity was defined as an absorbance change of 0.01 units/min.

### Superoxide dismutase assay (SOD)

SOD activity was estimated by the method of Kakkar *et al*. [15]. Reaction mixture of this method contained: 0.1 ml of phenazine methosulphate (186 μM), 1.2 ml of sodium pyrophosphate buffer (0.052 mM; pH 7.0), 0.3 ml of supernatant after centrifugation (1500 × g for 10 min followed by 10000 × g for 15 min) of testis homogenate was added to the reaction mixture. Enzyme reaction was initiated by adding 0.2 ml of NADH (780 μM) and stopped after 1 min by adding 1 ml of glacial acetic acid. Amount of chromogen formed was measured by recording color intensity at 560 nm. Results are expressed in units/mg protein.

### Glutathione-S-transferase assay (GST)

Glutathione-S-transferase activity was assayed by the method of Habig *et al*. [16]. The reaction mixture consisted of 1.475 ml phosphate buffer (0.1 mol, pH 6.5), 0.2 ml reduced glutathione (1 mM), 0.025 ml (CDNB) (1 mM) and 0.3 ml of homogenate in a total volume of 2.0 ml. The changes in the absorbance were recorded at 340 nm and enzymes activity was calculated as nM CDNB conjugate formed/min/mg protein using a molar extinction coefficient of 9.6 × 10^3^ M^-1^cm^-1^.

### Glutathione reductase assay (GR)

Glutathione reductase activity was determined by method of Carlberg and Mannervik [17]. The reaction mixture consisted of 1.65 ml phosphate buffer: (0.1 mol; pH 7.6), 0.1 ml EDTA (0.5 mM), 0.05 ml oxidized glutathione (1 mM), 0.1 ml NADPH (0.1 mmol) and 0.1 ml of homogenate in a total volume of 2 ml. Enzyme activity was quantitated at 25°C by measuring disappearance of NADPH at 340 nm and was calculated as nM NADPH oxidized/min/mg protein using molar extinction coefficient of 6.22 × 10^3^ M^-1^cm^-1^.

### Glutathione peroxidase assay (GPx)

Glutathione peroxidase activity was assayed by the method of Mohandas *et al*. [18]. The reaction mixture consisted of 1.49 ml phosphate buffer (0.1 M; pH 7.4), 0.1 ml EDTA (1 mM), 0.1 ml sodium azide (1 mM), 0.05 ml glutathione reductase (1 IU/ml), 0.05 ml GSH (1 mM), 0.1 ml NADPH (0.2 mM), 0.01 ml H_2_O_2_ (0.25 mM) and 0.1 ml of homogenate in a total volume of 2 ml. The disappearance of NADPH at 340 nm was recorded at 25°C. Enzyme activity was calculated as nM NADPH oxidized/min/mg protein using molar extinction coefficient of 6.22 × 10^3^ M^-1^cm^-1^.

### Quinone reductase assay (QR)

The activity of quinone reductase was determined by the method of Benson *et al*. [19]. The 3.0 ml reaction mixture consisted of 2.13 ml Tris–HCl buffer (25 mM; pH 7.4), 0.7 ml BSA, 0.1 ml FAD, 0.02 ml NADPH (0.1 mM), and 0.l ml of homogenate. The reduction of dichlorophenolindophenol (DCPIP) was recorded at 600 nm and enzyme activity was calculated as nM of DCPIP reduced/min/mg protein using molar extinction coefficient of 2.1 × 10^4^ M^-1^cm^-1^.

### Reduced glutathione assay (GSH)

Reduced glutathione was estimated by the method of Jollow *et al*. [20]. 1.0 ml sample of homogenate was precipitated with 1.0 ml of (4%) sulfosalicylic acid. The samples were kept at 4°C for 1 h and then centrifuged at 1200 × g for 20 min at 4°C. The total volume of 3.0 ml assay mixture contained 0.1 ml filtered aliquot, 2.7 ml phosphate buffer (0.1 M; pH 7.4) and 0.2 ml DTNB (100 mM). The yellow color developed was read immediately at 412 nm on a SmartSpecTM plus Spectrophotometer. It was expressed as μM GSH/g tissue.

### Estimation of lipid peroxidation assay (TBARS/LPO)

The assay for lipid peroxidation was carried out following the modified method of Iqbal *et al*. [21]. The reaction mixture in a total volume of 1.0 ml contained 0.58 ml phosphate buffer (0.1 M; pH 7.4), 0.2 ml homogenate sample, 0.2 ml ascorbic acid (100 mM), and 0.02 ml ferric chloride (100 mM). The reaction mixture was incubated at 37°C in a shaking water bath for 1 h. The reaction was stopped by addition of 1.0 ml 10% trichloroacetic acid. Following addition of 1.0 ml 0.67% thiobarbituric acid, all the tubes were placed in boiling water bath for 20 min and then shifted to crushed ice-bath before centrifuging at 2500 × g for 10 min. The amount of TBARS formed in each of the samples was assessed by measuring optical density of the supernatant at 535 nm using spectrophotometer against a reagent blank. The results were expressed as nM TBARS/min/mg tissue at 37°C using molar extinction coefficient of 1.56 × 10^5^ M^-1^cm^-1^.

### Hydrogen peroxide assay (H_2_O_2_)

Hydrogen peroxide (H_2_O_2_) was assayed by H_2_O_2_-mediated horseradish peroxidase-dependent oxidation of phenol red by the method of Pick and Keisari [22]. 2.0 ml of homogenate sample was suspended in 1.0 ml of solution containing phenol red (0.28 nM), horse radish peroxidase (8.5 units), dextrose (5.5 nM) and phosphate buffer (0.05 M; pH 7.0) and were incubated at 37°C for 60 min. The reaction was stopped by the addition of 0.01 ml of NaOH (10 N) and then centrifuged at 800 × g for 5 min. The absorbance of the supernatant was recorded at 610 nm against a reagent blank. The quantity of H_2_O_2_ produced was expressed as nM H_2_O_2_/min/mg tissue based on the standard curve of H_2_O_2_ oxidized phenol red.

### Molecular studies

DNA had been isolated and its fragmentation percent was quantified in molecular studies of *in vivo* toxicity.

### DNA fragmentation assay with diphenylamine reaction

DNA fragmentation from tissue extract was determined using the procedure of Wu *et al*. [23]. 100 mg tissue was homogenized in TTE solution. 0.1 ml of homogenate was labeled B, centrifuged at 200 × g at 4°C for 10 min, got supernatant labeled S. S tubes were centrifuged at 20,000 × g for 10 min at 4°C to separate intact chromatin, was labeled T. 1.0 ml of 25% TCA was added in all tubes T, B, S and incubated over night at 4°C. After incubation precipitated DNA was recovered by pelleting for 10 min at 18,000 × g at 4°C. 160 μl of 5% TCA was added to each pellet and heated for 15 min at 90°C then 320 μl of freshly prepared DPA solution was added, vortexed and incubated for 4 hr 37°C. Optical density was read at 600 nm with a spectrophotometer (Smart spec™ Plus, catalog # 170–2525).

### DNA Isolations and ladder assay

DNA was isolated by using the methods of Wu *et al.*[23]. 100 mg of tissue in a petri dish was washed with DNA Buffer and homogenized in 1 ml lysis buffer. 100 μl of proteinase K (10 mg/ml) and 240 μl 10% SDS, shaked gently, and incubate overnight at 45°C in a water bath then 0.4 ml of phenol, was added shaked for 5–10 min, and centrifuge at 3000 rpm for 5 min at 10°C. Supernatant was mixed with 1.2 ml phenol, 1.2 ml Chloroform/isoamyl alcohol (24:1); shaked for 5–10 min, and centrifuged at 3000 rpm for 5 min at 10°C. 25 μl of 3 M sodium acetate (pH 5.2) and 5 ml ethanol was added with supernatant, shake until DNA was precipitated. DNA was washed with 70% ethanol, dried, dissolved in TE buffer and its concentration checked at 260 and 280 nm. 5 μg of total DNA and 0.5 μg DNA standard per well were loaded on 1.5% agarose gel containing ethidium bromide. Electrophoresis was performed for 45 min with 100 V batteries, and DNA was observed under digital gel doc system and photographed.

### Histopathological study of tissue

After weighting the portion specifies for histology small pieces of each tissue was fixed for 3–4 h in fixative sera followed by dehydration with ascending grades of alcohol (80%, 90%, and 100%) and transferred in cedar wood oil. When tissue becomes clear then all tissues were embedded in paraplast and prepared blocks for further microtomy. 3–4 μm thin slides were prepared with microtome; wax was removed, stained with hemotoxilin-eosin and photographed under light microscope at 10x and 40x.

### Statistical analysis

To find the different treatment effects of *in vivo* studies one way analysis of variance was carried by computer software SPSS 13.0. Level of significance among the various treatments was determined by LSD at 0.05% level of probability.

## Results

### Phytochemical determination

#### High performance liquid chromatography (HPLC)

**Figure 1:**
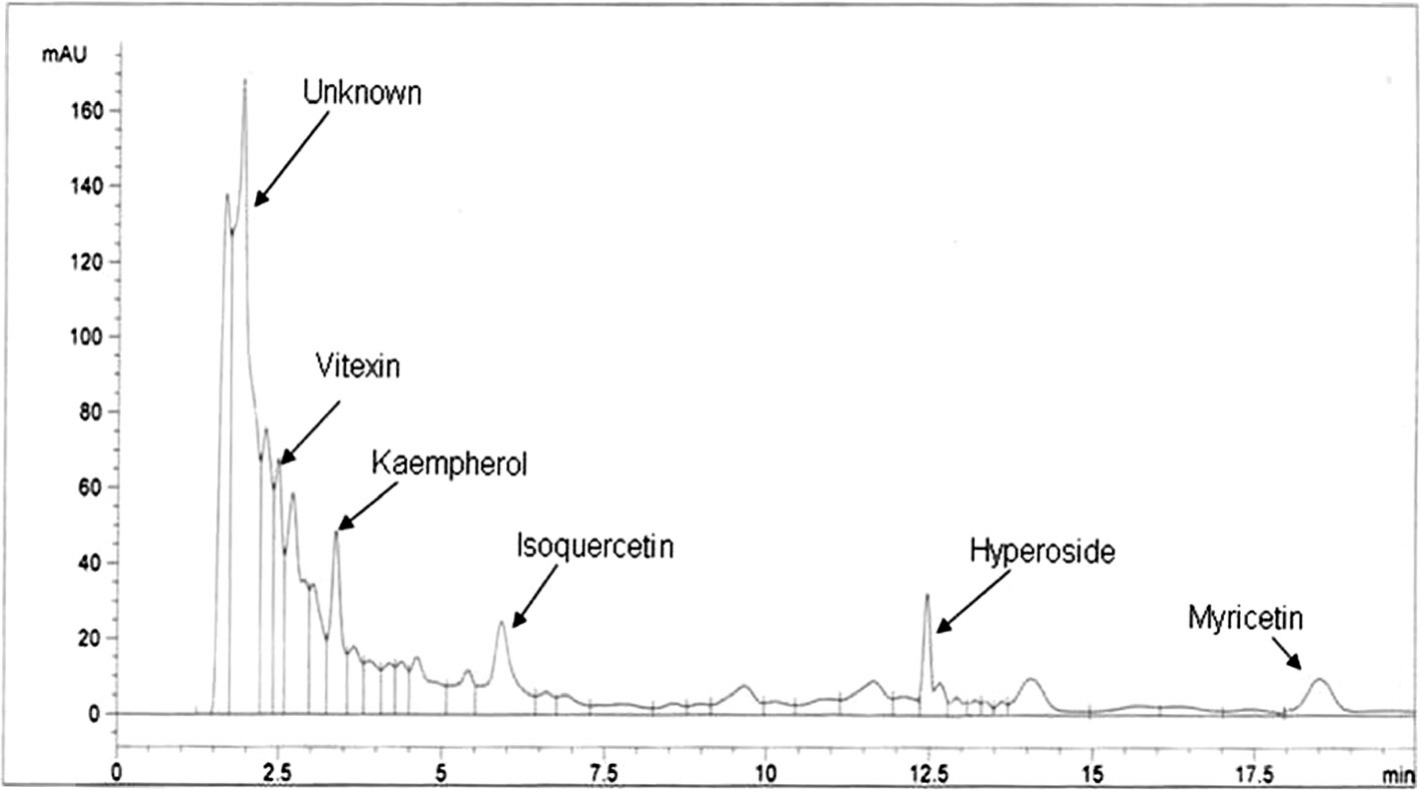
**1HPLC flavonoid profile of MLC, Conditions: mobile phase, ACN-dH**_**2**_**O; flow rate, 1 ml min**^**−1**^**; detection wave length, 220 nm; column temperature, 36°C; injection volume, 10 μl.**

In the present study, HPLC-UV was preferred for the qualitative as well as quantitative analysis of methanolic extract of *C. opaca* leaves. All experimental conditions were optimized to get the chromatograms with better resolution within a short resolution time and maximum UV absorption of sample and flavonoid standard compounds were quantified by assimilation of peak areas at 220 nm within runtime of 20 minutes as summarized in Table 1. The conditions used directed towards the good separation of peaks that may be identified in the chromatogram as apigenin (R_t_ =4.7), myricetin (R_t_ =18.5), vitexin (R_t_ =2.5), orientin (R_t_ =2.75), hyperoside (R_t_ =12.5), isovitexin (R_t_ =3.7), isoquercetin (R_t_ =6), rutin (R_t_ =8.7), luteolin (R_t_ =2.01), kaempherol (R_t_ =3.4), luteolin-7-glucoside (R_t_ =1.6). A sample of 10 μl of solution was injected to the instrument and identification was done by comparing the obtained peaks of chromatogram of samples with the peaks of standard flavonoids in respect to retention time and UV-spectra. The chromatogram determining flavonoids components of different fractions of *C. opaca* leaves in Figure 1. Table 2 summarized the flavonoids found in methanolic fraction of *C. opaca* leaves. There were some peaks having different retention time could not be identified; however, based on their chromatographic behaviors and UV spectra, they may correspond to unknown flavonoids compounds as presented in respective chromatogram.

**Table 1:** Linear regression analysis of eleven standard flavonoids

**Compound**	**Retention time**	**Regression analysis**	**R**	**Linear range (ppm)**
Rutin	8.7	y = 12571.3x-16.62	0.9873	10-250
Myricetin	18.5	y = 9643.4x-11.07	0.9919	10-200
Vitexin	2.5	y = 23085.1x + 3.72	0.9932	10-100
Orientin	2.75	y = 36421.0x + 2.88	0.9869	25-500
Hyperoside	12.5	y = 22758.9x + 1.56	0.9865	10-250
Isovitexin	3.7	y = 31604.2x + 0.98	0.9741	5-150
Luteolin	2.01	y = 19348.6x + 2.08	0.9532	5-100
Isoquercetin	6	y = 26785.6x + 1.60	0.9616	5-500
Apigenin	4.7	y = 10623.5x-9.82	0.9765	25-250
Kaempherol	3.4	y = 26182.8x- + 2.33	0.9417	10-500
Luteolin-7-glucoside	1.6	y = 11434.6x-10.72	0.9536	5-100

Values are Mean ± SD (03 number).

**Table 2:** **Assessment of flavonoids in methanolic extract of *Carissa opaca *leaves extract**

**Compound**	**Retention time**	**Concentration μg/mg of dry weight**
Isoquercitin	6	0.119
Hyperoside	12.5	0.062
Vitexin	2.5	0.053
Myricetin	18.5	0.172
Kaempherol	3.4	0.08

Values are Mean ± SD (03 number).

### *In Vivo* investigation

Previous studies reported that liver is not the just target organ of CCl_4_; it can actually distress other vital organs including kidney, lungs, heart and testis. Consequently, the intention of the current study was to assess the CCl_4_ as one of the male reproductive toxicant, hence alteration in the male reproductive hormones, antioxidant levels, lipid profile of serum and histopathological changes of the testis were investigated.

### Effects of MLC against CCl_4_ induced testicular toxicity in rat

The current study was paying attention on the estimation of ameliorating potential of *C. opaca* leaves against testicular toxicity provoked by CCl_4_. The biomarkers for testicular toxicity evaluation were based on serological studies, antioxidant enzyme levels of tissue, genotoxicity and histological variation of testis.

### Effects of MLC on male reproductive hormones of rats

Data from the serological markers for reproductive status such as testosterone, luteinizing hormone (LH), follicle stimulating hormone (FSH), prolactin and estradiol is summarized in Table 3. CCl_4_ intoxication drastically (p < 0.05) reduced the serum level of testosterone, LH and FSH, while notably (p < 0.05) increased the intensity of prolactin and estradiol. The serum level of above said reproductive hormones was re-established (p < 0.05) by oral administration of 100 mg/kg b.w., 200 mg/kg b.w., MLC near to control group. 50 mg/kg body weight of silymarin treatment erased CCl_4_ intoxication and restored the level of all tested reproductive hormones in serum of rats.

**Table 3:** Effects of MLC on male reproductive hormonal level

**Group**	**Testosterone**	**Luteinizing**	**Follicle Stimulating**	**Prolactin**	**Estradiol**
	**(ng/ml)**	**Hormone (ng/ml)**	**Hormone (ng/ml)**	**(ng/ml)**	**(ng/ml)**
Control	4.2 ± 0.07d	2.28 ± 0.09c	40.37 ± 0.14h	10.43 ± 0.34e	17.43 ± 0.32g
Oil + DMSO	4.1 ± 0.13d	2.23 ± 0.01c	39.12 ± 0.36h	11.23 ± 0.32e	15.24 ± 0.43g
CCl_4_	1.9 ± 0.08a	1.10 ± 0.08a	16.68 ± 0.21a	22.11 ± 0.44a	30.16 ± 0.65a
Sily + CCl_4_	3.5 ± 0.10c	1.89 ± 0.12b	32.87 ± 0.48g	14.4 ± 0.22d	20.23 ± 0.63f
100 mg/kg b.w. MLC + CCl_4_	2.2 ± 0.03c	1.74 ± 0.05b	23.04 ± 0.35c	16.01 ± 0.50c	24.18 ± 0.29c
200 mg/kg b.w. MLC + CCl_4_	3.30 ± 0.23c	2.01 ± 0.13b	28.15 ± 0.49f	16.23 ± 0.21c	22.43 ± 0.25d
MLC alone	4.0 ± 0.07d	2.36 ± 0.09c	40.85 ± 0.62h	12.27 ± 0.30e	14.37 ± 0.30g

Values are Mean ± SD (06 number), Sily = Silymarin.

a-f (Means with different letters) indicate significance at p < 0.05.

### Effects of MLC on lipid panel changes

Table 4 summarizes protective effects of MLC against CCl_4_ induced toxicity in lipids profile of serum. For lipid parameters total triglycerides, total cholesterol, HDL, and LDL were investigated.CCl_4_ disputation markedly increased the levels of triglycerides, total cholesterol, and LDL cholesterol while decreased (p < 0.01) HDL cholesterol as against the control group. Treatment of MCL cancelled the toxicity of CCl_4_ thus, restoring the serum level of total triglycerides, total cholesterol, HDL, and LDL towards the control group. Treatment with silymarin also produced similar results.

**Table 4:** Effects of MLC on lipid profile

**Group**	**Triglycerides (mg/dl)**	**Total Cholesterol (mg/dl)**	**HDL (mg/dl)**	**LDL (mg/dl)**
Control	130.13 ± 2.97e	39.15 ± 1.43e	32.31 ± 1.08d	15.34 ± 1.34d
Oil + DMSO	128.76 ± 2.45e	40.24 ± 1.34e	30.32 ± 1.73d	13.56 ± 1.11d
CCl_4_	230.19 ± 2.08a	78.99 ± 1.05a	52.36 ± 1.04a	30.61 ± 2.04a
Sily + CCl_4_	159.32 ± 2.42d	49.23 ± 2.33d	40.33 ± 1.27c	20.76 ± 1.73c
100 mg/kg b.w. MLC + CCl_4_	190.06 ± 4.41b	60.73 ± 1.72b	48.23 ± 1.12b	25.26 ± 1.95b
200 mg/kg b.w. MLC + CCl_4_	168.99 ± 2.22c	52.84 ± 2.62c	44.84 ± 1.69c	22.33 ± 1.41c
MLC alone	127.19 ± 1.33e	39.65 ± 1.28e	29.47 ± 1.36d	12.15 ± 1.11d

Values are Mean ± SD (06 number). Sily = Silymarin.

a-e (Means with different letters) indicate significance at p < 0.05.

### Effects of MLC on testis enzymatic antioxidant levels

Oxidative stress produced by CCl_4_ upsets the cellular antioxidant defense system. The protective effects of MLC against CCl_4_ toxicity on the antioxidant profile are presented in Table 5. Administration of CCl_4_ for eight weeks caused noteworthy (p < 0.05) decrease in the tissue soluble protein and CAT, POD and SOD activities as opposite to control group. Post-treatment of 100 mg/kg b.w., 200 mg/kg b.w., MLC markedly ameliorated the affects of CCl_4_ intoxication, and distinctly enhanced (p < 0.05) testicular protein and CAT, POD and SOD levels of testicular tissue. Lipid peroxidation is umpired via free radicals produced by CCl_4_ intoxication. The significant decline in H_2_O_2_ and TBARS level of testicular tissue corroborates with protective power 100 mg/kg b.w., 200 mg/kg b.w., MLC against testicular CCl_4_ induced lipid peroxidation in testis tissue_._ Similar protective effects were reported, while treating with silymarin.

**Table 5:** Effects of MLC on tissue proteins and antioxidant enzyme levels

**Group**	**Protein**	**CAT**	**POD**	**SOD**	**TBARS**	**H**_**2**_**O**_**2**_
	**(μg/mg tissue)**	**(U/min)**	**(U/min)**	**(U/mg protein)**	**(nM/min/mg protein)**	**(μM/ml)**
Control	1.421 ± 0.023d	3.92 ± 0.22d	9.38 ± 0.13c	3.08 ± 0.23d	2.97 ± 0.16d	1.123 ± 0.010c
Oil + DMSO	1.495 ± 0.026d	4.02 ± 0.55d	9.04 ± 0.34c	3.12 ± 0.14d	3.10 ± 0.27d	1.244 ± 0.012c
CCl_4_	0.834 ± 0.029a	2.02 ± 0.11a	4.76 ± 0.51a	0.88 ± 0.07a	5.71 ± 0.48a	2.536 ± 0.023a
Sily + CCl_4_	1.312 ± 0.008c	3.54 ± 0.34c	8.58 ± 0.38b	2.75 ± 0.07c	3.80 ± 0.33c	1.560 ± 0.091b
100 mg/kg b.w. MLC + CCl_4_	1.27 ± 0.062c	2.9 ± 0.17b	7.79 ± 0.85c	1.95 ± 0.13c	3.74 ± 0.70b	1.30 ± 0.069c
200 mg/kg b.w. MLC + CCl_4_	1.344 ± 0.073c	3.25 ± 0.32c	8.37 ± 0.41b	2.29 ± 0.11b	4.01 ± 0.25c	1.644 ± 0.065b
MFC alone	1.441 ± 0.012d	3.95 ± 0.68d	10.00 ± 0.61c	3.63 ± 0.13d	2.91 ± 0.33d	1.121 ± 0.045c

Values are Mean ± SD (06 number). Sily = Silymarin.

a-h (Means with different letters) indicate significance at p < 0.05.

Alterations in phase II antioxidant metabolizing enzymes viz; GST, GPx, GR, GSH and QR as well as DNA fragmentation% testicular tissues of rat are demonstrated in Table 6. Chronic administration of CCl_4,_ extensively (p < 0.05) abridged the glutathione status of GST, GPx, GR, GSH and QR whereas, percentage of DNA fragmentation was increased in comparison to non treated control group. Post-treatment with 100 mg/kg b.w., 200 mg/kg b.w., MLC attenuated the intoxication of CCl_4_ and restored the enzymes activity near to control rats. Silymarin treatment markedly lessened the DNA fragmentation% while, increased the GST, GSH, GR, GPx and QR activation similar to the effects of 100 mg/kg b.w., 200 mg/kg b.w., MLC.

**Table 6:** Effects of MLC on phase II antioxidant enzymes and DNA fragmentation

**Group**	**GST (nM/mg protein)**	**GPx (nM/mg protein)**	**GR (nM/mg protein)**	**GSH (μM/g tissue)**	**QR (nM/mg protein)**	**%DNA Injuries**
Control	150.23 ± 5.34g	110.17 ± 4.46f	198.34 ± 5.78e	16.25 ± 1.11c	99.90 ± 4.97f	14.53 ± 2.57f
Oil + DMSO	157.17 ± 4.45g	105.55 ± 4.55f	204.73 ± 6.47e	15.74 ± 1.31c	107.03 ± 4.42f	13.33 ± 2.24f
CCl_4_	78.37 ± 4.33a	63.33 ± 3.11a	103.14 ± 4.66a	9.60 ± 1.34a	52.40 ± 4.16a	57.50 ± 3.34a
Sily + CCl_4_	133.67 ± 2.43f	98.46 ± 2.22e	179.56 ± 4.45d	13.75 ± 0.32b	89.14 ± 2.56e	20.18 ± 3.73e
100 mg/kg b.w. MFC + CCl_4_	120.54 ± 1.98c	77.39 ± 2.92b	166.07 ± 2.21c	20.05 ± 0.90c	75.25 ± 3.12d	33.30 ± 0.25d
200 mg/kg b.w. MFC + CCl_4_	124.12 ± 2.81e	94.65 ± 2.16e	170.68 ± 4.97d	13.29 ± 0.46b	82.56 ± 3.67d	23.56 ± 2.36e
MFC alone	159.31 ± 3.21g	112.40 ± 3.26f	201.25 ± 4.12e	17.59 ± 1.45c	109.34 ± 2.23f	12.55 ± 1.27f

Values are Mean ± SD (06 number). Sily = Silymarin.

a-f (Means with different letters) indicate significance at p < 0.05.

### Effects of MLC on DNA damages (ladder assay)

**Figure 2:**
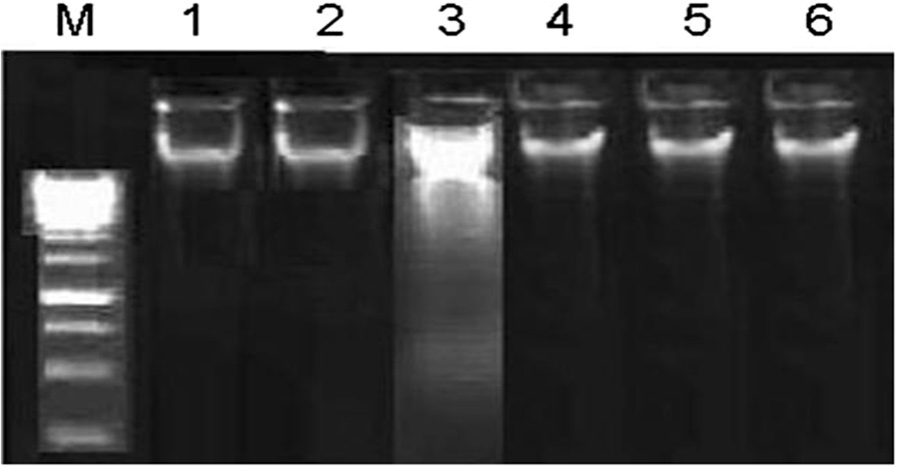
**Agarose gel showing DNA damage by CCl**_**4 **_**and protective effects of MLC in testicular tissue.** Lanes from left (**M**) low molecular weight marker, (**1**) control, (**2**) DMSO + Olive oil group, (**3**) CCl_4_ group, (**4**) Silymarin + CCl_4_ group, (**5**) 100 mg/kg b.w. MLC + CCl_4_ group, (**6**) 200 mg/kg b.w. MLC + CCl_4_ group.

Protective effects of different doses of MLC versus CCl_4_ induced DNA damage in the testicular tissues of rats is shown by DNA ladder assay in Figure 2. Extensive DNA breakages in testis were portrayed by the treatment of CCl_4_ to rats. Post-administration of silymarin and MLC prevented the DNA damages induced by CCl_4_ indicating the protective effects of *C. opaca* leave.

### Effects of MLC on testis histoarchitecture

**Figure 3:**
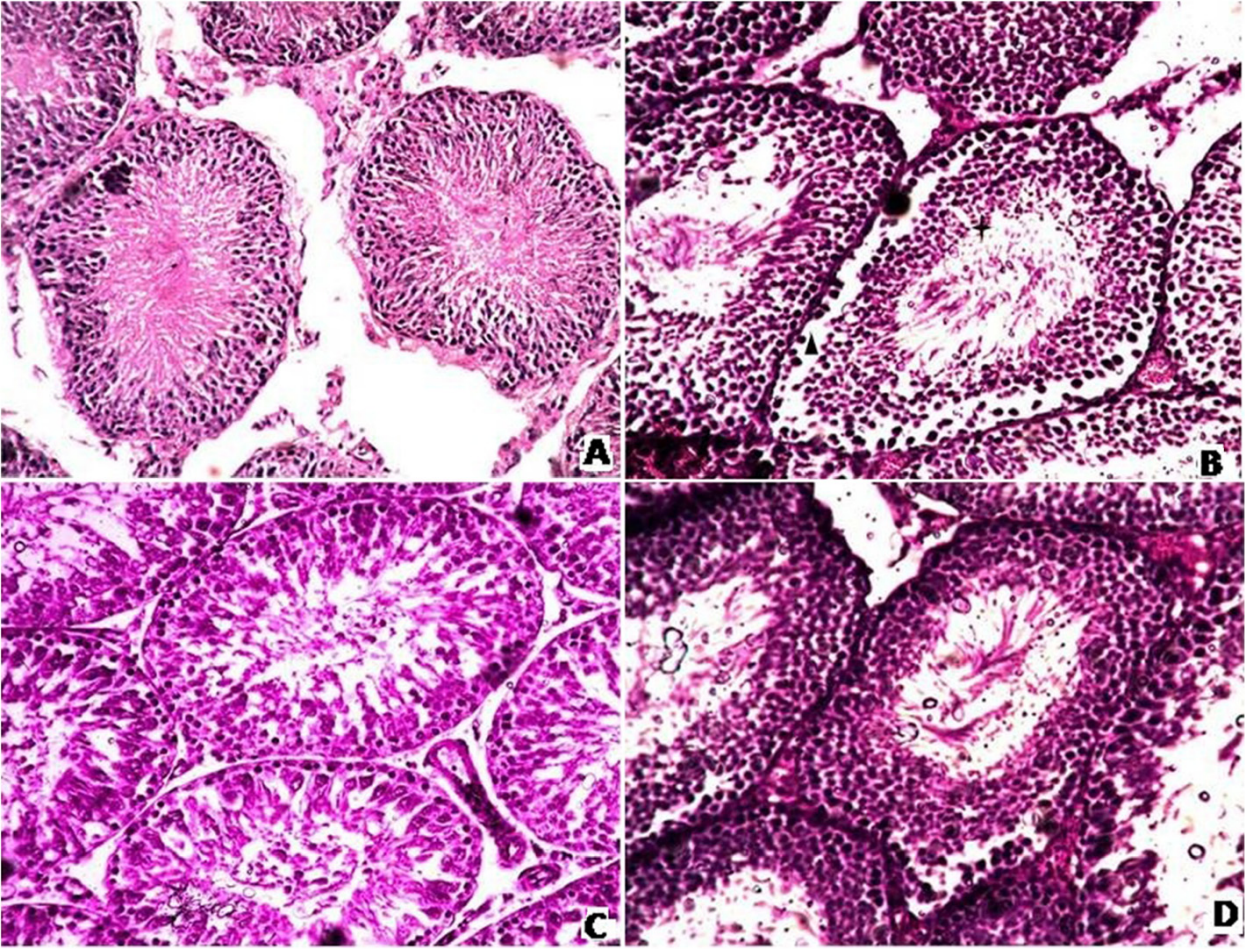
**Microphotograph of rat testis (H & E stain).** (**A**) Representative section of testis from the control group showing normal histology, (**B**) CCl_4_ group, (**C**) Silymarin + CCl_4_ group, (**D**) 200 mg/kg b.w. MLC + CCl_4_ group (✦) distorted and less concentration of germ cells, (▲) displaced interstitial cells away from the seminiferous tubules.

The histoarchitecture of testis after different treatments is presented in Figure 3. Light microscope evaluation of H & E stain showed normal testicular architecture possessing normal seminiferous tubules, normal concentration of germ cells, sperms with normal morphology and concentration and inconspicuous sertoli cells (Figure 3A). The CCl_4_ intoxication resulted in ruthless testicular injuries with imperative decrease in germ cells, vacuolization of germinative epithelium and dislocated interstitial cells away from basement membrane and seminiferous tubules as shown in Figure 3B. The silymarin group also showed almost normal structure of testis as compare to CCl_4_ intoxicated group (Figure 3C). Groups treated MLC showed improved concentration of sperms and stabilization of organized seminiferous tubules (Figure 3D). The results obtained from histological architecture were in consistency with the hormonal studies as well as antioxidant status.

## Discussion

Oxidative stress induced by an increase in free radicals and/or decrease in antioxidant defenses is well documented in animal model [24,25]. CCl_4_, a typical toxic agent, exerts its toxic effects by the generation of free radicals. By the activation of liver cytochromes P450, CCl_4_ is converted into free radicals which immediately react with cell membrane [26]. This free radical not only targets liver but it can also causes free radical generation in other tissues like kidneys, heart, lung, testis, brain and blood [27,28]. In the current study, the proposed plan aimed to assess and examine the possibility of MLC to protect and reduce the lipid peroxidation and oxidative damages caused by CCl_4_ in testis tissue homogenate of male Sprague Dawley rats. The reduction of testosterone levels in serum indicates either direct effects of CCl_4_ at Leydig cell level or indirect effects by disturbing the hormonal environmentat hypothalamo-pituitary axis [29] due to oxidative trauma in CCl_4_ treated rats. It was reported that abnormal level of intra testicular testosterones inhibits spermatogenesis [30]. Tohda *et al.* (2001). The production of testosterone in Leydig cells is stimulated by LH, which activates FSH to bind with sertoli cells to stimulate spermatogenesis [31]. CCl4 intoxicated rats show the malfunctioning of pituitary to secrete FSH and LH indicating testicular dysfunction leading to infertility as was reported by previous results [32]. GSH levels are dependent upon the activities of glutathione reductase (GR) and NADH [33]. Glutathione system including GPx, GR, GST, as well as SOD and CAT represent a mutually loyal team of defense against ROS [34]. Enhanced lipid peroxidations expressed in terms of TBARS determine structural and functional alterations of cellular membranes [35]. In the present study, administration of various fractions of plant samples in different experimental groups improved the activities of SOD, CAT, POD, GPx, GST, GR and QR as well as non enzymatic (GSH, TBARS and H_2_O_2_) levels of CCl_4_-intoxicated testis towards normalcy in warfare of oxidative trauma *in vivo*. Hence, the present results regarding chronic toxicity of CCl_4_ are in accordance with previous reports [36,37], while studying the protective effects of *Sonchus asper* and *Launeae procumbens* on testis against oxidative stress of CCl_4_. Present study revealed that the activities of antioxidant enzymes were significantly reduced the toxication of chemical which might be due to the presence of bioactive elements like myricetin, kaempherol, isoquercetin, hyperoside and vitexin, propagating free radicals like peroxyl radicals and converting the reactive free radicals to inactive products.

It was reported that CCl_4_ resulted in the oxidative damage to testicular proteins in rats. Oxidative damage to proteins is very important as it can contribute secondary damage resulting in hampering the DNA repair enzymes and loss of reliability of damage polymerases during DNA replication. The DNA damage in various tissues like brain, testis and liver was reported by Manierea *et al*. [38]. From the present study, it can be assumed that chronic exposure of CCl_4_ may cause accumulation of many toxic species in cells thus damaging both DNA and lipids. In fact, treatment with various fractions of plant samples ameliorated the toxic effects on DNA as revealed by DNA fragmentation % and ladder assay. The present study clearly augments the defensive mechanism of various samples against oxidative stress induced by CCl_4_ and provides confirmation about its therapeutic use in reproductive abnormalities.

Previous studies on histomorphology of testis showed shrinkage of the tubular diameter and testicular atrophy leading to degenerative changes in the germinal epithelium [39] after exposure to toxic chemical. Similar destructive effects were also accounted in CCl4 treated groups. The CCl_4_ challenge revealed testicular destruction and degeneration in histological architecture like that of profenofos that was recorded by Moustafa *et al*. [40] who represented damaged columnar epithelial layer, vacuolated spermatogonial cells, oedematous alterations in the seminiferous tubules and extra elongated Leydig cells. Data of the present study revealed that CCl_4_ may hamper continuing proliferative behavior of testicular cells thus obstruct reproduction. Deformities in spermatogenesis and partial degeneration of germ and Leydig cells have been displayed by CCl_4_-treated rats. However, groups administered various fractions of plant samples in different experiments demonstrated a quality active spermatogenesis, thin basement membranes and normal seminiferous tubules in most of the part of testis. Same histopathology was noticed by Manjrekar *et al*. [41], while evaluating the protective effects of *Phyllanthus niruri* Linn treatment on testis against CCl4 intoxication. This paper is in continuation of our previous studies Sahreen *et al.*[42] in which hepatoprotective effect of methanolic extract of leaves were asasessed.

## Conclusion

It can be concluded from the current study that bioactive components of MLC especially flavonoids (myricetin, kaempherol, isoquercetin, hyperoside and vitexin) have the ability to recover the metabolic enzymatic activities and repair cellular injuries, thus providing scientific evidence in favor of its pharmacological use in testicular dysfunctioning.

## Competing interest

The authors declare that they have no competing interests.

## Authors’ contributions

SS made a significant contribution to acquisition of data, analysis, drafting of the manuscript. MRK and RAK have made a substantial contribution to conception and design, interpretation of data, drafting and revising the manuscript for intellectual content. All authors read and approved the final manuscript.
